# Microbial Metabolite Urolithin B Inhibits Recombinant Human Monoamine Oxidase A Enzyme

**DOI:** 10.3390/metabo10060258

**Published:** 2020-06-19

**Authors:** Rajbir Singh, Sandeep Chandrashekharappa, Praveen Kumar Vemula, Bodduluri Haribabu, Venkatakrishna Rao Jala

**Affiliations:** 1 Department of Microbiology and Immunology, James Graham Brown Cancer Center, University of Louisville, Louisville, KY 40202, USA; rajbirphd83@gmail.com (R.S.); haribabu.bodduluri@louisville.edu (B.H.); 2 Institute for Stem Cell Science and Regenerative Medicine (inStem), University of Agricultural Sciences-Gandhi Krishi Vignan Kendra (UAS-GKVK) Campus, Bellary Road, Bangalore, Karnataka 560065, India; sandeepc@instem.res.in (S.C.); praveenv@instem.res.in (P.K.V.)

**Keywords:** microbial metabolites, urolithins, monoamine oxidase

## Abstract

Urolithins are gut microbial metabolites derived from ellagitannins (ET) and ellagic acid (EA), and shown to exhibit anticancer, anti-inflammatory, anti-microbial, anti-glycative and anti-oxidant activities. Similarly, the parent molecules, ET and EA are reported for their neuroprotection and antidepressant activities. Due to the poor bioavailability of ET and EA, the in vivo functional activities cannot be attributed exclusively to these compounds. Elevated monoamine oxidase (MAO) activities are responsible for the inactivation of monoamine neurotransmitters in neurological disorders, such as depression and Parkinson’s disease. In this study, we examined the inhibitory effects of urolithins (A, B and C) and EA on MAO activity using recombinant human MAO-A and MAO-B enzymes. Urolithin B was found to be a better MAO-A enzyme inhibitor among the tested urolithins and EA with an IC_50_ value of 0.88 µM, and displaying a mixed mode of inhibition. However, all tested compounds exhibited higher IC_50_ (>100 µM) for MAO-B enzyme.

## 1. Introduction

Monoamine oxidases (MAO, EC 1.4.3.4) are flavoproteins located in the outer mitochondrial membrane of neuronal and non-neuronal cells. These enzymes are responsible for the oxidative deamination of monoamines (e.g., serotonin, dopamine, methylhistamine and tryptamine) in CNS as well as in peripheral sites. The MAO enzymes exist in two forms, MAO-A and MAO-B, and share 70% similarity at the amino acid level. Besides sharing 70% identity, they possess specific selectivity towards substrates and inhibitors [1]. MAO isoforms are expressed ubiquitously; however, their expression levels vary among different tissues. In humans, MAO-A is highly expressed in sympathetic nerve terminals, placenta and intestinal mucosa, which helps in the deamination of exogenous monoamines. In contrast, MAO-B is highly expressed in the liver, platelets, skeletal muscles and brain tissues [2]. The MAOs are critical to maintain the homeostasis of biogenic amines, such as dopamine and norepinephrine in the brain. Therefore, any alteration in their activities would eventually lead to several neurological and psychiatric disorders. The MAO enzymes have been targeted to alleviate various neurological disorders. Clinically, MAO-A inhibitors are used for the treatment of depression and anxiety [3], while MAO-B inhibitors are used for slowing down the progression of Parkinson’s disease [4].

ET and EA are major polyphenolics in pomegranate, walnuts and berries. The consumption of ET-rich diets has been acknowledged for various health benefits over the last few decades. Although ETs are not absorbed in the gastrointestinal tract, the in vivo conversion of ETs to EA, followed by the gut microbial metabolism of EA, produce urolithins, which have higher absorption and bioavailability [5]. Urolithins are generated from EA by opening and decarboxylating one of the lactone rings and subsequent sequential removal of hydroxyl groups (as reviewed in [6]). Previously, it was shown that the consumption of pomegranate juice or EA improved the depression stage (acting as anti-depressants) in mouse models potentially through the monoaminergic system [7,8]. Due to the poor absorption and bio-availability of EA, it is not clear whether the anti-depressant effects are mediated by EA itself or by their gut microbial metabolites, urolithins. Recently, it was shown that urolithins can inhibit β-amyloid fibrillation (in vitro) and protect Aβ1−42-induced neurotoxicity and paralysis in *Caenorhabditis elegans* [9]. Therefore, we hypothesized that the observed beneficial neurological effects rendered by EA and pomegranate juice are due to inhibitory effects mediated by urolithins on MAO enzymes. We tested this hypothesis by examining the direct effects of urolithins (A, B and C) and EA on MAO-A and MAO-B enzyme activities.

## 2. Materials and Methods

### 2.1. Chemicals and Reagents

Human recombinant MAO-A and MAO-B enzyme isoforms were purchased from Sigma-Aldrich (St. Louis, MO, USA). A MAO-Glo™ Assay kit (V1401) was purchased from Promega Corporation (Madison, WI, USA). Chlorgylline (MAO-A inhibitor) and deprenyl (MAO-B inhibitor) compounds were purchased from Santa Cruz Biotechnology (Dallas, TX, USA). All other chemicals and reagents were HPLC grade or the highest purity available from Sigma-Aldrich (USA) or VWR International (Radnor, PA, USA).

### 2.2. Monoamine Oxidase Assay, Determination of IC_50_ and Evaluation of Inhibition Kinetics

MAO inhibition assays were performed using a MAO-Glo™ Assay kit (Promega) according to the manufacturer’s instructions. Briefly, 1 µg of human recombinant MAO-A and MAO-B enzyme isoforms were incubated with 160 and 16 µM of MAO substrates, respectively. The enzyme reactions were performed in the presence or absence of EA or urolithin-A (UA), urolithin-B (UB) or urolithin-C (UC) in a 96-well white plate (Thermo Fisher Scientific, Waltham, MA, USA) at 37 °C for 1 h. The enzyme reaction was quenched by the addition of a luciferin detection reagent. The standard inhibitors of MAO-A (chlorgylline; 1 μM) and MAO-B (deprenyl; 5 μM) were used as positive controls. An equal volume of dimethyl sulfoxide (0.1%) was added to the enzyme assay as a control. After 1 h of incubation, the luminescence was measured with a multimode microplate reader (Synergy HT, BioTek, Winooski, VT, USA) to determine the MAO enzyme activities. Initially, the inhibition potential of all the test compounds was evaluated at a concentration of 100 μM. The control reaction (with no inhibitor) with vehicle (DMSO 0.1%) was set at 100% activity. To determine IC_50_ of the test compounds, the dose dependent enzyme assays were performed. We used range of 0.1 to 50 μM concentrations for MAO-A and 0.1 to 200 μM concentrations of test compounds for the MAO-B enzyme activities. The type of enzyme inhibition was determined using variable concentrations of MAO-A substrate (10–360 µM) and MAO-B substrate (2–64 µM) for the respective enzyme assay. The enzyme assays were performed in the presence of UA (3, 12 μM), UB (0.4, 2 μM) or UC (15, 60 μM). A Lineweaver–Burk double reciprocal plot was generated, and the mode of inhibition was determined by visual examination of the plot. The slope of the reciprocal plots was plotted against the inhibitor concentrations and determined the x-axis intercept that represented the inhibitor constant, Ki value.

### 2.3. Data Analysis

All experiments were performed in triplicates and data are presented as mean ± standard error of the mean (SEM). The raw data are provided in Supplementary Data, Table S1. Enzyme kinetic data were analyzed using the Lineweaver–Burk plot. Statistical analysis was performed by Student’s *t*-test using GraphPad Prism Ver. 7.0 (GraphPad Prism Software Inc., San Diego, CA, USA).

## 3. Results and Discussion

**Figure 1:**
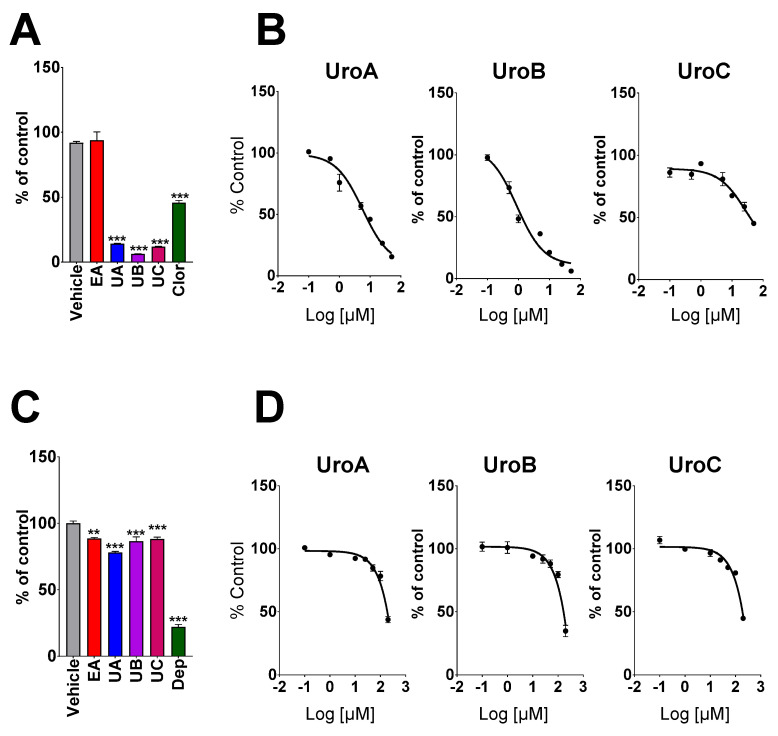
Effect of ellagic acid and urolithins on MAO activity. Ellagic acid, UroA, UroB and UroC (100 µM; primary screening) effect on MAO-A activity (**A**) and MAO-B (**C**). Dose-dependent (0–50 µM for MAO-A and 0–200 µM for MAO-B) effect of Uro A, B and C on MAO-A activity (**B**) and MAO-B enzyme activity (**D**). Chlorgylline (1 µM) and deprenyl (5 µM) were used as positive control. Asterisks *** represent *p* < 0.001, ** *p* < 0.01 compared to vehicle.

**Figure 2:**
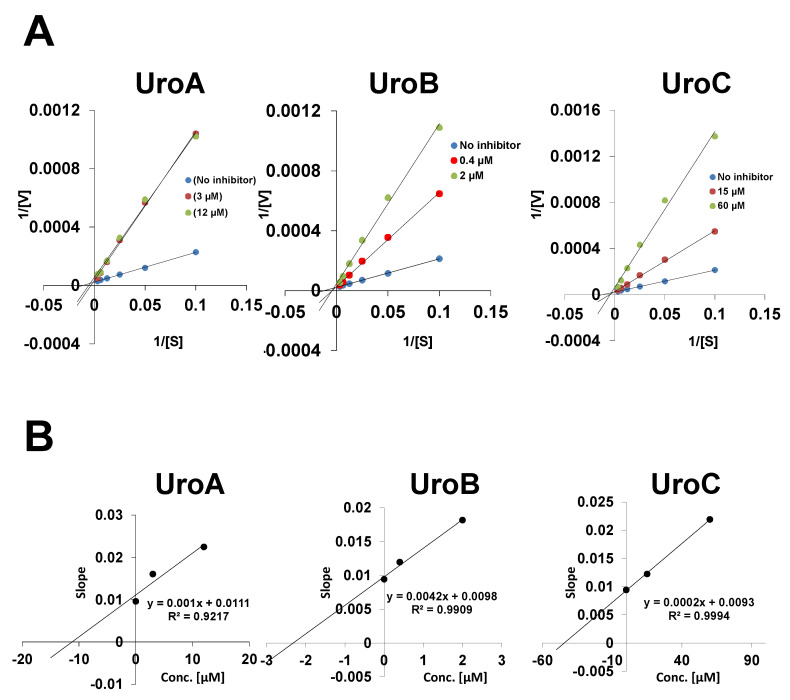
Lineweaver–Burk plot of UroA (3 and 12 µM), UroB (0.4 and 2 µM) and UroC (15 and 60 µM) for MAO enzyme activity (**A**), and slopes of Lineweaver–Burk plots were used to determine Ki values (**B**).

The present study evaluated the effects of EA and its gut microbiota-derived metabolites (UA, UB and UC) on MAO enzyme activities. Treatment with UA, UB and UC (100 µM) significantly inhibited the MAO-A enzyme activity by 86%, 94% and 88%, respectively, compared to the vehicle. EA failed to inhibit MAO-A enzyme activity. Chlorgylline (1 μM), a positive control, inhibited 55% of MAO-A activity, as shown in Figure 1A. IC_50_ values of UA, UB and UC for MAO-A inhibitory activity were determined using a range of concentrations (0–50 μM). We observed a dose-dependent inhibitory effect of UA, UB and UC with IC_50_ of 5.88 ± 0.69, 0.88 ± 0.24 and 29.6 ± 1.8 μM, respectively. The Lineweaver–Burk plot of UA, UB and UC for MAO-A suggested the mixed mode of inhibition with a Ki value of 10, 2.3 and 44.3 µM, respectively, as shown in Figure 2. However, all test compounds (EA, UA, UB and UC) exhibited only 20–30% inhibition for MAO-B activity, even at higher concentrations (100 μM) tested. The positive control, deprenyl (5 μM), showed more than 60% inhibition of MAO-B enzyme activity, as shown in Figure 1C. 

The inhibitors of MAO, such as selegiline (Emsam), isocarboxazid (Marplan), phenelzine (Nardil), and tranylcypromine (Parnate), are being used in the clinic to treat depression and anxiety [10,11]. Previous studies have suggested that pomegranate juice, ellagic acid and urolithins mediate some of the antidepressant effects in animal models [5,7,8,9]. However, mechanisms of action are yet to be determined. Here, we postulated that urolithins, the biologically active metabolites of EA, are responsible for the reported neuroprotective activities of EA and pomegranate juice by inhibiting MAO enzyme activities. Compared to EA/ET, urolithins have a much higher rate of absorption in the intestines and exert numerous biological activities [6,12,13]. It is pertinent to point out that urolithins can be detected up to micromolar concentrations in some individuals, based on the presence of appropriate microbiota and their consumption of ET/EA-rich diets, such as pomegranate, berries, walnuts, etc. [14,15,16]. Considering the low bioavailability of ET and EA, the in vivo antidepressant effects of these compounds could be attributed to their gut microbial metabolites and urolithins, which could block MAO-A enzyme activity.

MAO-B inhibitors are currently being used to treat the symptoms of Parkinson’s disease (PD) in humans. In a recent study, the administration of pomegranate juice prevented PD-like characteristics in rats. Importantly, UA was detected in the brains of these rats, suggesting the ability of UA to cross the blood-brain barrier and potentially mediating neuroprotective activities against PD [17]. We cannot rule out the possibility of the presence of other urolithins, as these studies were only targeted to detect UA. We also observed the low (<10–20%), but significant inhibition of MAO-B activity by urolithins at higher doses, which could also contribute to the observed in vivo beneficial activities of pomegranate juice in rat PD models. It is possible that urolithins exert beneficial activities through multiple pathways by protecting against oxidative damage and α-synuclein aggregation [17], as well as the inhibition of MAO-B activities, leading to the mitigation of PD symptoms in these rats. The urolithins selectively inhibited MAO-A enzyme activities with higher efficacies compared to the MAO-B enzyme.

The substrates and inhibitors of MAO bind to the cavity which is occupied by Flavin. The binding site in MAO-A is present in a single-chamber, while the MAO-B binding site is bifurcated, containing the outer entry chamber and inner combining site. Entry to the MAO-B active site (combining site) is hindered due to the presence of ile199, however, this restriction is relieved with the binding of a suitable substrate [18]. The small entrance cavity in MAO-B only permits small hydrophobic molecules, and larger hydroxylated compounds are not permitted due to their size and charge. It is possible that entry of the urolithins could be responsible for the differential inhibitory activities on MAO-A and MAO-B enzymes. Therefore, these selective inhibitory activities could be due to the comparatively large entry site of MAO-A enzyme, which is amenable to urolithins, whereas the entry site in MAO-B is hindered by the presence of ile199 [18,19]. It is possible that EA is a larger molecule than urolithins and thus may not gain entry access to inhibit MAO enzyme activity. These predictions require further investigations through computer modeling and the crystal structures of MAO with substrates.

## 4. Conclusions

Our studies demonstrated that Urolithin B is a potent inhibitor of MAO-A among UA, UB, UC, and their parent compound, EA. Considering the fact that urolithins can cross the blood-brain barrier [9], these studies could provide a missing link for the known beneficial effects of pomegranate juice or EA in mitigating symptoms of depression. To the best of our knowledge, this is the first study that highlights the direct inhibitory potential of urolithins on MAO enzyme activities. Furthermore, the benefits of urolithin-mediated MAO inhibitory activities require in-depth investigations, utilizing pre-clinical models to prove their pharmacological and clinical significance. This study could be of greater importance in the alleviation of neurological disorders, especially depression, as these compounds are demonstrated to cross the blood-brain barrier without exhibiting any known toxicities.

## Supplementary Materials

The following are available online at https://www.mdpi.com/2218-1989/10/6/258/s1, Table S1: The original replicative data set points used to generate the graphs.
